# CircRNAs in Malignant Tumor Radiation: The New Frontier as Radiotherapy Biomarkers

**DOI:** 10.3389/fonc.2022.854678

**Published:** 2022-03-16

**Authors:** Xixi Wu, Junying Wu, Lingxia Wang, Wei Yang, Bo Wang, Huan Yang

**Affiliations:** ^1^ Department of Clinical Laboratory, The Second Affiliated Hospital of Soochow University, Suzhou, China; ^2^ Department of Clinical Laboratory, The Children’s Hospital of Soochow University, Suzhou, China; ^3^ State Key Laboratory of Radiation Medicine and Protection, Soochow University, Suzhou, China; ^4^ Department of Oncology, The Second Affiliated Hospital of Soochow University, Suzhou, China

**Keywords:** circRNAs, radiotherapy, malignant tumor, biomarkers, clinical application

## Abstract

World Health Organization (WHO) data show that of the top 20 factors that threaten human life and health, cancer is at the forefront, and the therapeutic approaches for cancer consist of surgery, radiotherapy, chemotherapy and immunotherapy. For most highly metastatic and recurrent cancer, radiation therapy is an essential modality to mitigate tumor burden and improve patient survival. Despite the great accomplishments that have been made in clinical therapy, an inevitable challenge in effective treatment is radioresistance, the mechanisms of which have not yet been completely elucidated. In addition, radiosensitization methods based on molecular mechanisms and targets, and clinical applications are still inadequate. Evidence indicates that circular RNAs (circRNAs) are important components in altering tumor progression, and in influencing resistance and susceptibility to radiotherapy. This review summarizes the reasons for tumor radiotherapy resistance induced by circRNAs, and clarifies the molecular mechanisms and targets of action. Moreover, we determine the potential value of circRNAs as clinical indicators in radiotherapy, providing a theoretical basis for circRNAs-based strategies for cancer radiotherapy.

## Introduction

Radiation therapy is one of the conventional methods in cancer treatment. It is utilized in more than 50% of oncology patients and can be applied alone or in combination with immunotherapy and chemotherapy (1). Radiation-induced DNA double-strand breaks are one of the most lethal causes of cell death. When exposed to radiation, DNA double-strands break directly or cells generate surplus free radicals that indirectly damage DNA (2). Synchronously, radiotherapy can also induce apoptosis, autophagy and cell cycle changes, thereby altering tumor cell proliferation, invasion and other properties. However, there are still some unavoidable problems in radiotherapy, for example, how to reduce side effects and implement precision radiotherapy strategies. Of these problems, radiation tolerance is a common and intertwined phenomenon that impedes therapeutic efficacy, resulting in the neoplasm recrudescence or poor prognosis after radiotherapy (3, 4). Consequently, it is of great significance to uncover the mechanisms of radiation resistance, predict sensitivity to radiotherapy in patients and formulate strategies to overcome radioresistance.

CircRNAs, a naturally occurring event of widespread and multitudinous single-stranded RNAs, were initially misinterpreted as useless products of splicing in the 1970s. Subsequently, their structures and functions have been broadly expounded by high-throughput RNA sequencing. The distribution of circRNAs is diverse, with a small fraction located in the nucleus, and they intervene in the transcription of parental genes by RNA polymerase II (5) or U1 small nuclear ribonucleoprotein (snRNP) (6). UAP56 and URH49 are responsible for the nuclear transport of circRNAs (7), and these molecules transported into the cytoplasm act as competing endogenous RNAs (ceRNAs) by sponging miRNAs. Accumulating evidence demonstrates that circRNAs have emerged as one of the central regulators of human diseases (8). In tumors, circRNAs are linked to radiation resistance and radiosensitivity. When receiving radiation, altered circRNAs regulate the radiotherapy response by activating signal pathways and targeting genes, inducing cellular process changes, such as DNA damage repair (DDR), epithelial–mesenchymal transition (EMT), apoptosis, autophagy, cell cycle, metabolism (9).

Previous studies have stated that dysregulated circRNAs are involved in chemotherapy (10, 11), showing good prospects and clinical value. However, the current systematic generalization of circRNAs in radiotherapy is limited. Here, we describe the origin and characterization of circRNAs, and systematically review the regulation mechanisms of circRNAs in radiotherapy. We also discuss targeted therapy strategies and other potential clinical values of circRNAs in irradiation (IR).

## Backgrounds of circRNAs: Classification, Biogenesis and Features

### Classification and Biogenesis

**Figure 1:**
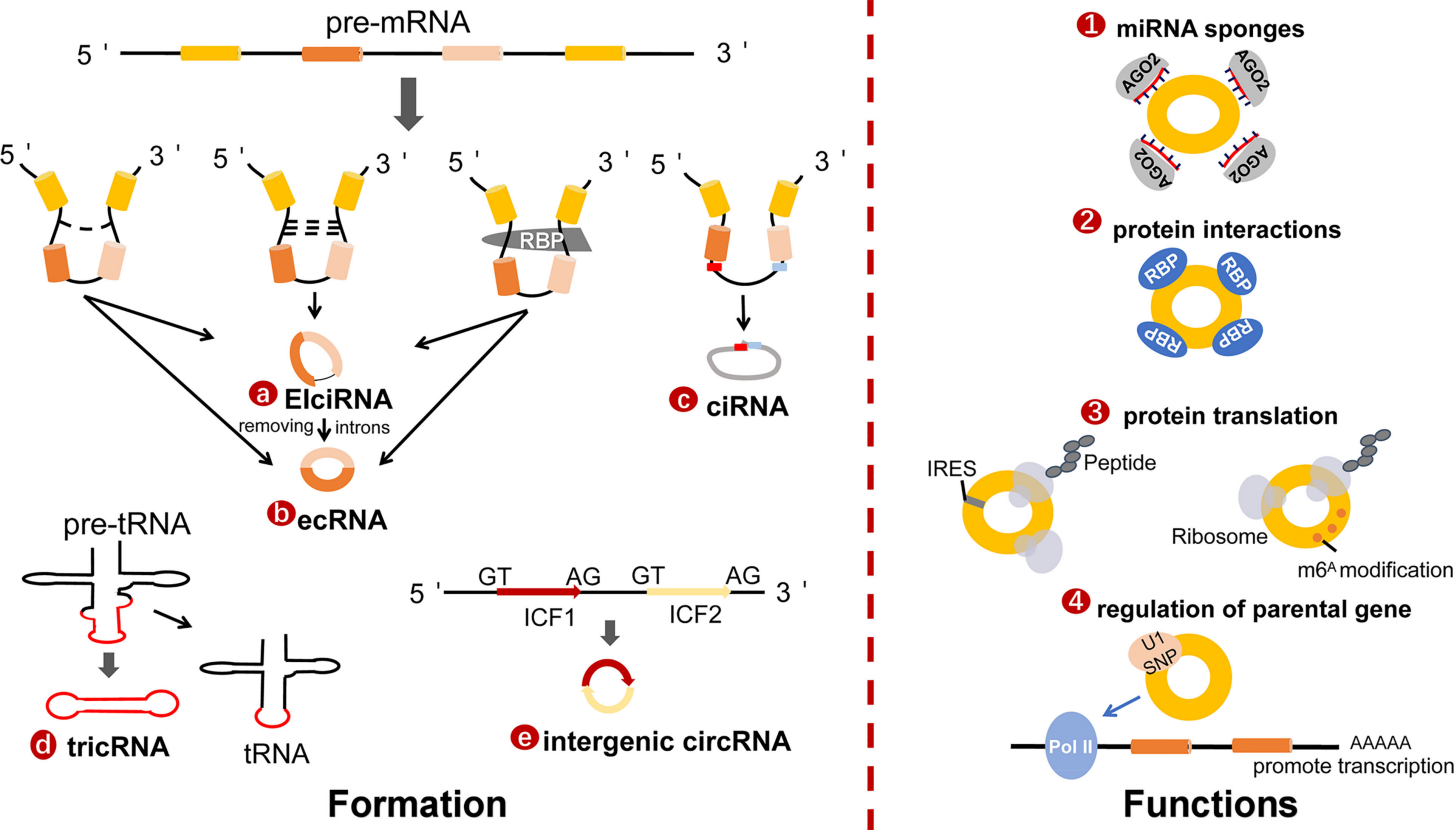
The formation and functions of circRNAs. **(A)** Exon-intron circRNAs (EIciRNA) are the nuclear retained and promote the transcription of parental genes. **(B)** Exonic circRNAs (ecRNAs) are generally supported as products of canonical spliceosome. When pre-mRNA splicing removes introns while retaining exons, the downstream 3′splicing site binds to upstream 5′splicing site in reverse order. **(C)** Intron circRNAs (ciRNAs). The sequence close to the 5′ splicing site containing a 7 nt GU-rich element (red box) and an 11 nt C-rich element near the branch point (blue box) are essential to stable ciRNAs. **(D)** TRNA intronic circular RNAs (tricRNAs), the production of tricRNAs requires conserved tRNA sequence and several processing enzymes, such as RtcB ligase and TSEN endonuclease. **(E)** Intergenic circRNA. Intron-containing fragments (ICFs) flanking GT-AG splicing signals, acting as splice donors and splice acceptors to conduct intergenic circRNAs through circularization. The main functions of circRNAs include miRNA sponges, protein interactions, protein translation and regulation of parental gene.

CircRNAs, single-stranded covalently closed continuous loop RNA without a 5′ cap and 3′ poly A tail, were first reported in models of viroid in 1976 (12). Subsequently, advanced bioinformatic technology identified and classified different types of circRNAs, which are ubiquitous in various cells and tissues (13). These circRNAs are also unique in terms of their origin and structure. Previous studies have identified the following four categories based on various combinations of exons and/or introns (14): (1) Exonic circRNAs (ecRNAs) are generally thought to be products of canonical spliceosomes. When pre-mRNA splicing removes introns and retains exons (15, 16), the downstream 3′ splicing site binds to the upstream 5′ splicing site in reverse order (17). Several studies have suggested that exon-only circRNAs can act as regulators in the cytoplasm (18); (2) Exon-intron circRNAs (EIciRNAs), which are nuclear retained and can promote the transcription of parental genes (6); (3) Intron circRNAs (ciRNAs), unlike most introns that are degraded immediately after excision, some introns have the capacity to escape branching and form circRNAs containing introns instead (19, 20). CiRNAs predominantly exist in the nuclear region, indicating that they may interact with host gene transcription (19); (4) Transfer RNA intronic circular RNAs (tricRNAs). Pre-tRNA removes 5’ leader and 3’ trailer by tRNA splicing endonuclease, and then modifies the CCA structure on 3 tail ends to form mature tRNA. RtcB ligase is not only responsible for the splicing of tRNA, but also the circularization process of tricRNAs (21, 22). Interestingly, some scholars discovered a new type of circRNAs named intergenic circRNAs. Intron-containing fragments (ICFs) flanking GT-AG splicing signals, act as splice donors and splice acceptors to conduct intergenic circRNAs through circularization (23). In **Figure 1**, we briefly describe the formation and functions of circRNAs, whose functions are determined by their sequence, post-transcriptional modification, and location. Commonly proposed functional mechanisms of circRNAs include miRNA sponges, protein interactions, translation, and regulation of parental genes.

The regulation mechanisms engaged in the formation of circRNAs transcripts include cis- and trans-acting factors (24). One of the essential cis-acting elements is the external reverse complementary sequences flanking exon splicing introns (25). Alu repeats (short repetitive sequence), the first determined elements that modulate back-splicing by tightly connecting to the splice sites (26), result in multiple circRNAs produced from a single gene locus (25). The existence of Alu repeats in flanking introns is an important basis for predicting and analyzing the formation of circRNAs (25). Additionally, RNA-binding proteins (RBPs) act as activators or inhibitors to regulate the production of circRNAs, respectively. Studies have showed that some RBPs can bind to intronic regions and improve back-splicing efficiency by homo- or hetero-dimerization (27, 28) to facilitate circularization. For instance, Sam68 can interact with the Alu-rich introns in the survival of motor neuron gene (SMN) pre-mRNA to promote circRNAs biogenesis (29). On the other hand, a few proteins may inhibit the formation of circRNAs by disrupting the base pairing of introns. Adenosine-to-inosine(A-to-I) edited by adenosine deaminase acting on RNA 1 (ADAR1) antagonizes circRNAs expression through weakened inverted Alu repeats (30). Similarly, nuclear RNA helicase DHX9 threatens circRNAs processing by untwisting the reverse complementary of Alu elements flanking exons (31).

### The Features of circRNAs

The alternative translation of the same genetic locus produces multiple RNA isoforms, endowing the isoforms with unique functions (32). CircRNAs and homologous linear mRNAs are derived from the same splicing precursor. Compared with the linear mRNAs, circRNAs not only have special structures, but also have independent characteristic. (1) stability: The spliced products of precursor RNAs include linear RNAs, circular RNAs and others, of which linear RNAs are completely degraded, whereas lariat and circRNAs can resist RNase R-digestion (33), owing to the lack of accessible ends. Yehoshua et al. confirmed that the great majority of circRNAs were long-lived compared with linear mRNAs (34), as the half-life of circRNAs exceed 48 h while that of linear is less than 20 h (26); (2) abundance: Next-generation RNA sequencing corrected the early false assumption that circRNAs were errors with non-function and low expression during transcription. Numerous studies have been devoted to considering the expression and function of circRNAs (26, 30, 35, 36). Generally, the expression level of most circRNAs is only 5-10% of its linear transcript (37). However, some circRNAs possess special features. In human fibroblasts, more than 14% of transcribed genes have the ability to express circRNAs, under certain circumstances, which are more abundant than the corresponding mRNAs (26). Julia Salzman et al. clarified approximately 50 circRNAs that were highly expressed compared with their parental linear transcripts in all tested cell lines (38), especially in neuronal organs (28, 39, 40). Moreover, the relative percentage of circRNAs to their canonical linear RNAs shows a higher average in human blood (41). Analysis of circRNAs genome characteristics documented that alternative back-splicing (ABS) allows a single gene to produce diverse circRNAs with the same reverse splicing site (42). This may be one of the reasons for the diversity and specificity of circRNAs; (3) unique function: CircHIPK3 is derived from exon2 of the HIPK3 gene and impairs human cell growth while being silenced. However, the linear molecule HIPK3 mRNA has no similar biological functions (43). A genome-wide analysis of 144 prostate cancer specimens identified 7,232 circRNAs; 11.3% of which were highly expressed and were related to cell proliferation; approximately 90% of their linear counterparts were not necessary for proliferation (44). In addition, circRNAs and mRNA sometimes exhibit antagonistic relationships. The Zbtb7a gene generates coding mRNAs and exerts its tumor suppressive role, but the non-coding product of Zbtb7a, circPOK, acts as an oncogene in mesenchymal cancers (32). The E-cadherin variant protein (C-E-Cad) encoded by circ E-cadherin represents antithetical functions with E-cadherin. C-E-Cad accelerates glioma stem cell tumorigenicity through the independent activation of the oncogenic epidermal growth factor receptor (EGFR) signal pathway and EGFRviii by the exceptional C -terminal (45). Studies have shown that the association between circRNAs and parental gene is weak, suggesting that circRNAs are largely independent and not just byproducts of aberrant splicing. In a word, the unique expression pattern of circRNAs suggest that they likely possess functional significance.

## The Role and Mechanisms of circRNAs in Radiobiology

**Figure 2:**
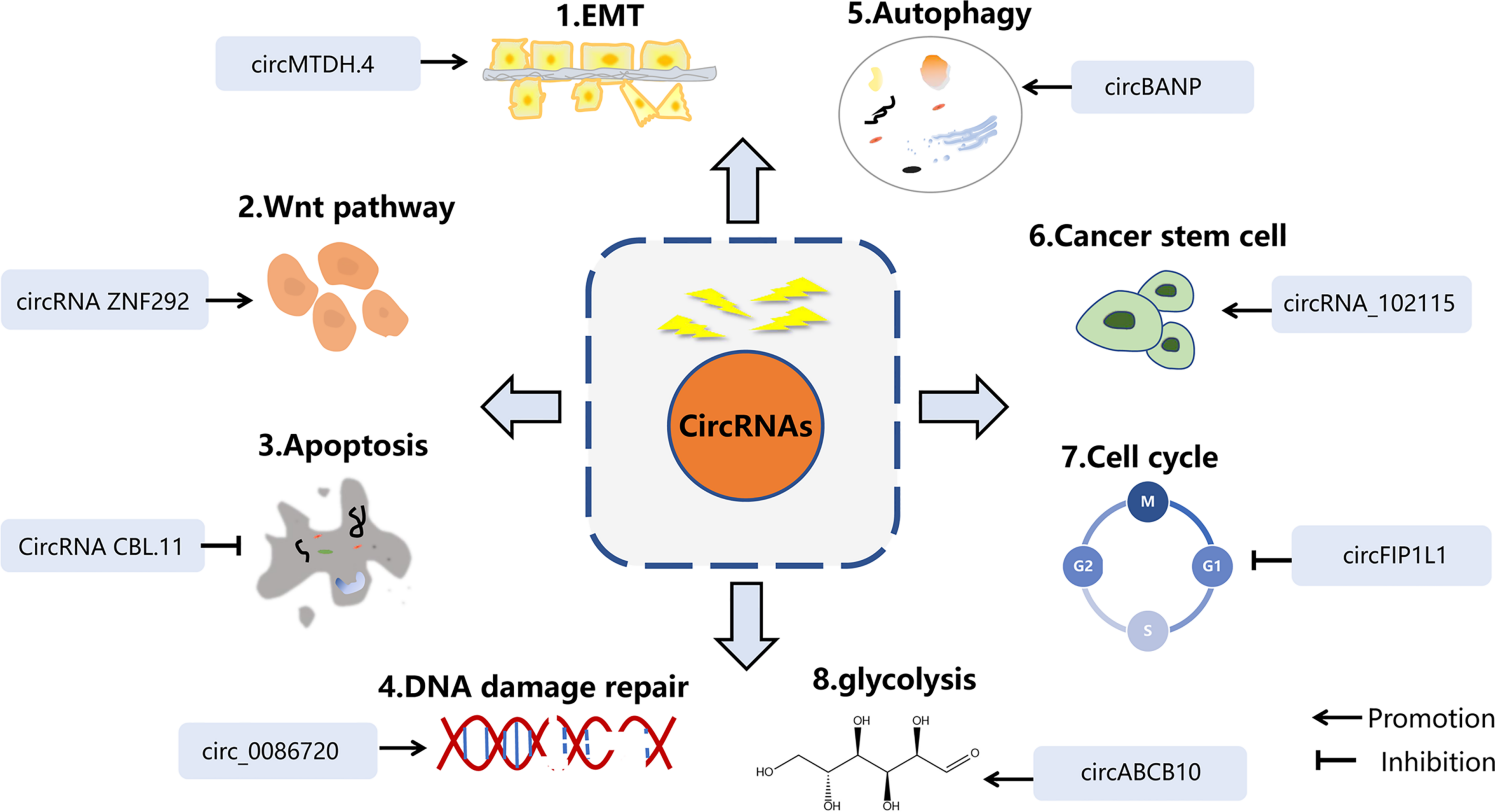
The mechanisms of circRNAs in radiobiology. The main mechanisms of cicRNAs in radiation inculde EMT, Wnt signaling, apoptosis, DNA damage repair, autophagy, cancer stem cell, and cell cycle arrest.

CircRNAs have emerged as significant regulators in the progress of tumors. Radiation is a linchpin of cancer treatment, which can alleviate sufferings or even completely cure tumor patients. Notwithstanding the advances in circRNAs research, the hidden mechanisms of how circRNAs contribute to radiotherapy remain largely unexplored. Here, we generalized the current mechanisms of action of circRNAs in modulating radiotherapy **(** **Figure 2** **)**, aiming to foster new insights into circRNAs therapeutic strategies.

### CircRNAs Participating in DNA Damage Repair

**Figure 3:**
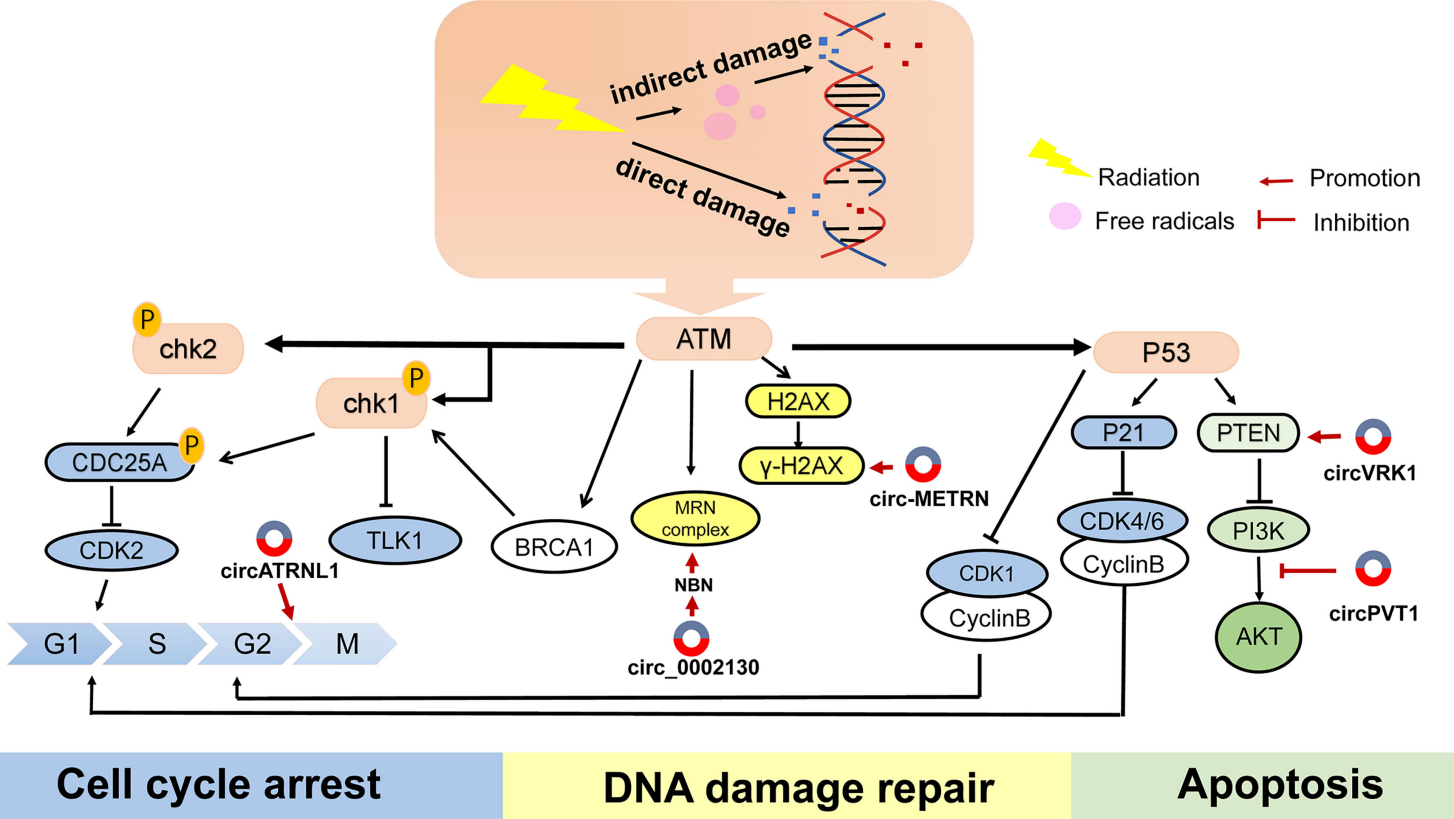
The main reaction of cells to DNA damage and the mechanism of several circRNAs on radiotherapy. DNA double-strand breaks (DSBs) are most cytotoxic injuries in respond to radiation, leading to cells death. When receiving radiotherapy, cells produce a series of repair mechanisms to deal with these damages, resulting in resistance to radiation.

Correct DNA repair is one of the basic methods of maintaining cell homeostasis. Inducing DNA strand damage is the pivotal biological function of radiotherapy, while activating the DNA-repair signaling pathway may attenuate the efficacy of anticancer therapy. ATM, ATR kinase, and DNA-PK are the key indicators in detecting DNA damage. Cell cycle checkpoint kinases CHK1, CHK2 are phosphorylated and activated by ATM and ATR after DNA damage, and play an important role in S phase and G2 phase (46). It has been proved that ATM-, ATR-, and DNA-PK inhibitors benefit tumor sensitivity to IR (46). In addition, γH2AX, TP53BP1 (47), and RAD51 (48) have been identified as sensitive candidates to predict radiation damage and repair. In particular, ATM has a central role in the perception of DNA damage and initiates a series of responses to cell cycle activation and apoptosis **(** **Figure 3** **)**.

Zinc finger E-box binding homeobox 1 (ZEB1) is an EMT-inducing transcription factor, and a well-known DDR regulator, responsible for chemo- or radio-resistance (49). Previous studies have shown that ZEB1 participated in radiation response with ATM kinase. For example, miR-875-5p increased radiation reaction by suppressing ZEB1, which impeded CHK1-mediated DNA homologous recombination repair (50). Similarly, down-regulation of circZEB1 reduced ZEB1 protein expression, thereby inhibiting CHK1 protein (51).

H2AX is a member of the histone H2A family. Within a few minutes after DNA double-strand breaking, H2AX is phosphorylated by ATM, ATR and DNA-PKcs to form γH2AX, which then rapidly recruits DNA repair proteins and apoptotic proteins to the injury site. H2AX phosphorylation modification is one of the cellular DNA double-strand break stress responses, and is also the most prominent DNA-associated marker (52). When absorbed in radiation, numerous circRNAs predispose the formation of γH2AX and maintain DNA repair, which is a critical component of radioresistance in cancer. Several circRNAs were reported to respond to IR-mediated H2AX regulation. Research has disclosed that inhibiting some circRNAs could effectively reduce the recruitment of γH2AX, thereby improving the sensitivity of radiotherapy. Si-circ-METRN (53) in glioblastoma as well as si-circ_0086720 (54) in non-small cell lung cancer (NSCLC) reversed circRNAs-mediated γH2AX activation. Mechanically, circ-METRN was regulated *via* the miR-4709-3p/GRB14/PDGFRα interaction network and circ_0086720 depletion reinforced radiation sensitivity by regulating the miR-375/SPIN1 axis. Conversely, circ-AKT3 overexpression increased the number of H2AX foci. As one of the AKT transcript variants, circ-AKT3 was under-expressed in glioblastoma and had the ability to encode AKT-174aa protein. AKT thr-308 phosphorylation was blocked by AKT-174aa, which interfered with downstream signal transmission. AKT-174aa acted as a negative regulator of the PI3K/AKT pathway and ultimately increased the radiosensitivity of glioblastoma multiforme (55). This research innovatively discovered the protein translation function of circRNAs. Although generally classified as non-coding RNAs, there is growing evidence that circRNAs have the ability to translate proteins. To sum up, delineation of exact signals which induce DDR during tumor treatment will undoubtedly help to provide a broader picture of how circRNAs exert their action.

### CircRNAs Are Involved in IR-Mediated Apoptosis

Apoptosis is proactive spontaneous death that maintains the stability of the intracellular environment. One of the main tasks of apoptosis is cleaning up precancerous cells and arresting the development of malignancy (56). However, dysregulation of apoptosis not only leads to unchecked cell proliferation and tumor occurrence, but also resistance to therapy. Therefore, the modulation of apoptosis signaling pathways is one of the key factors in optimizing cancer treatment.

The intrinsic apoptotic pathway is started by intracellular signals at the mitochondrial level in order to overcome various stresses, such as radiation and chemotherapy. Activation of the apoptotic pathway is closely associated with the B-cell lymphoma 2 family, which contains pro-apoptotic proteins (Bax) and anti-apoptotic proteins (Bcl-2). P53, a classic tumor suppressor gene, inhibits Bcl-2 activity by increasing the transcriptional expression of pro-apoptotic proteins Bax and Bak, thereby promoting apoptosis. In addition, the caspases are a family of cysteine-dependent endoproteases that regulate cell apoptosis (57). In this regard, several circRNAs have been proved to participate in IR-mediated apoptosis by affecting the above-mentioned pathways. CircRNA CBL.11 modulated YWHAE and varied equally to YWHAE in colorectal cancer cell (CRC) under carbon ion irradiation (58). A previous study reported that YWHAE was conducive to P53 signal activation. The up-regulated YWHAE indeed increased P53-mediated Bax expression and promoted apoptosis. However, this study only briefly introduced that circRNACBL.11 is involved in IR-mediated apoptosis, and functional research is still unclear.

PI3K/AKT signaling represents one of the most influential signaling pathways that inhibit cell apoptosis and promote cell survival. The high activation of PI3K/AKT is related to radiotherapy tolerance in various cancer types (59). The expression of circPVT1 in NSCLC cells following radiotherapy was higher than that previous to radiation. Down-regulated circPVT1 promoted apoptosis by blocking the PI3K/AKT/mTOR pathway and improved the radiosensitivity (60). The above studies further consolidated the role of circRNAs in radiotherapy.

Phosphatase and tension homology on chromosome 10 (PTEN) acts as a tumor suppressor, and governs a plethora of cellular processes, such as survival, proliferation, apoptosis. PTEN antagonizes PI3K activity by dephosphorylating PIP3 and inhibits the PI3K/AKT pathway, thereby preventing IR-mediated cell apoptosis. CircVRK1 heightened radiation sensitivity by adjusting the miR-624-3p/PTEN axis and inactivating the PI3K/AKT signaling pathway by upregulating PTEN (61). CircATRNL1 decreased in oral squamous cell carcinoma (OSCC) patients treated with radiation. Over-regulated circATRNL1 intensified OSCC radiosensitivity by directly binding to miR-23a-3p and relieving endogenous inhibition of the target gene PTEN, which is essential for apoptosis and cell-cycle arrest. Furthermore, adjustment of the PI3K/Akt signaling pathway was accompanied by the alteration of circATRNL1 (62). However, it is not yet certain whether the PI3K/Akt signaling pathway is a key process in circATRNL1 affecting OSCC radiotherapy. These corresponding results show that circRNAs can be utilized as a therapeutic target of radiation-mediated apoptosis in cancer.

### CircRNAs Regulate the Autophagy Response to Radiotherapy

Autophagy is a process which maintains cell homeostasis and responds to various stresses. When the cell is under external pressure, it will initiate an autophagy program against the damaged intracellular substances (63, 64). During this process, some damaged proteins or organelles are encapsulated by autophagic vesicles with a double-layer membrane structure, and then transferred to lysosomes for degradation and recycling. Accruing reports show that circRNAs facilitate radiotherapy and chemotherapy tolerance by inducing autophagy (65, 66). The crosstalk between circRNAs and radiation-mediated autophagy provides a new strategy for the regulation of tumors.

Microtubule-associated protein light chain 3 (LC3) and p62 play essential roles in the formation of autophagy. circRNA_102115 promoted the insensitivity of CRC through the endogenous competitive combination of miR-338-3p, increasing the level of LC3II/I and reducing p62 (67). Correspondingly, circCCNB2 knockdown inhibited autophagy of prostate cancer cells by up-regulating p62 but reducing Beclin1 and LC3II/I, thus increasing radiosensitivity. This effect relied on an interaction between circCCNB2 and miR-30b-5p as shown by rescue experiments. KIF18A was identified as a direct target of miR-30b-5p, and its ectopic expression restored radioresistance and restrained autophagy upon miR-30b-5p overexpression (68). From the current reports, different kinds of circRNAs exert different roles in tumor. Some inhibit autophagy and some promote autophagy. This shows that multiple circRNAs are involved in autophagy, but it is necessary to further clarify whether more circRNAs are participated in autophagy-related radiotherapy regulation. In the future, targeting autophagic pathway may become a new and potential mean of changing radiosensitivity.

### CircRNAs Adjust the Cell Cycle

Cell cycle regulation, a large and sophisticated network involving multiple factors, is essential in order to maintain normal cell growth. It includes cyclins and cyclin-dependent kinases (CDKs) that drive the cell cycle, as well as the brake system that exists to avoid unrestricted proliferation, such as CDKs inhibitory proteins (CDKIs) (69). Following damage of DNA molecules by radiotherapy (RT), related genes initiate cell cycle regulation mechanisms and retard cell cycle progression at the G1/S and G2/M checkpoints, with the G2/M period being the most sensitive to radiation, while the S phase exhibits radiation resistance. Cell cycle arrest enables damaged DNA more time to repair, or induces apoptosis on damaged cells that cannot be repaired, thus escaping IR killing effects and increasing radiation resistance (70). Several studies have shown that circRNAs affect cell cycle progression by alternatively binding to cell cycle proteins or acting on miRNA to regulate proteins. A study found that, compared with normal cells, the expression of circFIP1L1 decreased and the proportion of S phase increased in 5-8F-IR cells (radiotherapy-resistant nasopharyngeal cancer cell lines). Overexpression of circFIP1L1 rendered nasopharyngeal carcinoma (NPC) cells more sensitive to RT and resulted in accumulation of the cell cycle in G2/M phase (71). This effect on radiation and the cell cycle was mediated by the miR-1253/SFN pathway. SFN plays a negative role in the cell cycle and blocks the cell cycle at G2/M, acting as a key protein that maintains the stability of the genome (72). Similarly, in circATRNL1-increased OSCC cells, the percentage of G2 was higher and intensified OSCC radiosensitivity (61). CircRNA_014511 overexpression blocked the G2 phase and down-regulated the expression of P53 by coexisting with miR-29b-2-5p, affecting the cell cycle and apoptosis of bone marrow mesenchymal stem cells, and reducing sensitivity to RT (73). Inhibition of circPRKCI repressed viability, colony formation, cell cycle progression of esophageal cancer and elevated cell radiosensitivity through the miR-186-5p/PARP9 axis (74), showing that circPRKCI played a suppressor role in RT of esophageal cancer. As to how these circRNAs influence the biological effects of radiation, how they interfere with their expression to benefit clinical RT, etc., are attracting the attention of researchers.

### CircRNAs Manipulate Wnt Pathway in IR-Response

Wnt/β-Catenin, is an indispensable signaling pathway controlling development, differentiation, homeostasis, and stemness of tissues, and is closely associated with cancer progression. Further, a growing body of evidence has demonstrated that Wnt/β-Catenin signaling pathway in clinical therapy is a critical modulator driving cell phenotypic resistance to various types of anticancer treatment (75). The expression level of circRNAs in tumor cells changes after IR, which helps to initiate or inhibit the Wnt signaling, thereby altering the sensitivity of tumor cells to radiotherapy. For instance, circRNA-microarray identified 57 increased circRNAs and 17 decreased circRNAs in radioresistant esophageal cancer cell (ESCC) compared with the parental cell line. KEGG and GO analysis found that Wnt signals may be related to radioresistance (76). In this study, circRNA microarray was used to detect differentially expressed circRNAs, providing a basis for radiotherapy biology. CircRNA ZNF292 can be induced in hypoxia-responsive human hepatoma cell. By interfering with the nuclear translocation of SOX9 protein, circRNA ZNF292 eventually activated the Wnt signaling pathway and led to radioresistance (77). Knockdown of circRNA ZNF292 caused cell cycle arrest, proliferation inhibition, decreased angiogenesis and increased DNA fragmentation levels, accompanied by a decrease in Wnt/β-catenin pathway-associated proteins, including β-catenin, adenomatous polyposis coli (APC), and axis inhibition (Axin). Equally, the Wnt signaling pathway was found to be implicated in the regulation of circRNA_100367, with a higher expression of circRNA_100367 was observed in radiation-resistant ESCC cells (78). These studies suggest that there is a close relationship between circRNAs, Wnt/β-catenin signaling and RT, and extensive testing is needed to reveal the mechanism of circRNAs in radiotherapy-related Wnt/β-catenin signaling.

### CircRNAs Affect EMT in Radiobiology

Epithelial–mesenchymal transition is a biological process in which epithelial cells transform into cells with a mesenchymal phenotype, and plays a key role in cell biological behavior. After tumor cells are irradiated, the cells lose polarity, as well as tight junctions and adhesion. The invasion, migration and carcinogenicity of tumor cells are enhanced, and the ability of cells to resist apoptosis is also increased. Li et al. discovered that circMTDH.4/miR‐630/AEG‐1 axis contributed to the improvement of radioresistance in NSCLC cells. Previous research illustrated that AEG-1 induce EMT remolding by Wnt/β‐catenin signaling pathway. Li et al. indeed confirmed that AEG-1 silencing blocked Slug and Snail gene. In addition, knocking down circMTDH.4 or overexpressing miR‐630 can improve radiosensitivity (79). In line with this, deprivation of circRNA_100367 enhanced the radiation sensitivity of radioresistant ESCC cells through competitively binding to miR-217/Wnt3 axis and mediating EMT process. CircRNA_100367 silencing increased E‐cadherin, accompanied by a decrease in mesenchymal markers (78). Taking into consideration the conclusions presented, it is clear that circRNAs are involved in the EMT process as biomarkers of radiation response in different cancer types.

### CircRNAs Modulate Glycolysis in Response to IR

Glycolysis is a universal method of carbohydrate catabolism in all life. In glycolysis, glucose is degraded to generate ATP, which provides energy for organisms. Studies have indicated that the accumulation of lactic acid is an indication for tumor development, and the aggravation of IR resistance in tumor cells (80). Compared with normal tissues and cells, circABCB10 was up-regulated in breast cancer (BC) samples. When circABCB10 was silenced, the proliferation, colony formation and radioresistance of BC cells were limited. This was because it sponged miR-223-3p to regulate profilin-2 (PFN2), exerting a facilitating role on glycolysis and contributing to the enhancement of glucose consumption and lactate production (81). A similar effect was shown in glioma cells where circPITX1 knockdown decreased glucose consumption and lactate production through the miR-329-3p/NEK2 axis, thereby increasing the sensitivity to RT (82). From the evidence shown above, it is therefore plausible that glycolysis might be the major reason for the decreased benefit of RT. This conclusion opens new avenues to better understand the hidden aspects of circRNAs modulating glycolysis in response to IR.

### CircRNAs Influence Cancer Cell Stemness

Cancer stem cells (CSCs) are a group of heterogeneous cells with stem cell characteristics in the tumor. CSCs are endowed with the ability to self-renewal and infinite proliferation; thus, they can evade effective treatment and develop antitumor properties (83). Zhu et al. found that CD133+ cells increased after exposure to radiation in NPC CNE-2 cells, suggesting tumor stem-like cells were associated with NPC radiotherapy. They hypothesized that some dysregulated circRNAs induced NPC cells to differentiate into stem cells after irradiation. Bioinformatics suggested that the “hsa_circRNA_102115-hsa-miR-335-3p-MAPK1” interaction network was associated with CSCs, thus changing radiation sensitivity (84). The CSC-mediated mechanisms of radiotherapy resistance are multifactorial, including high DNA damage repair ability, cell cycle arrest, autophagy. Unfortunately, Zhu et al. have not yet clarified which mechanism CSC plays in NPC. The presence of CSCs has been largely demonstrated in therapy resistance. However, information on the involvement of circRNAs in CSCs research is scarce. In future, we may be able to focus more attention on the relationship between circRNAs and CSCs.

### CircRNAs Are Linked to the Tumor Microenvironment

The tumor microenvironment (TME) is the location between tumor cells and adjacent normal tissues. It is mainly composed of tumor cells, surrounding stromal cells and infiltrating inflammatory cells. Emerging evidence shows that external pressure has the ability to remold physiological conditions and metabolism of cancer and stromal cells. Altering the TME may have an impact on radiotherapy outcome (85).

Inflammatory cells or cytokines are in involved in the formation of inflammatory TME. Inflammation has been shown to be a risk factor for the occurrence and development of cancers. Radiation can induce DNA damage while stimulating the release of pro-inflammatory mediators and remolding the tumor immune microenvironment (86). To date, two circular RNAs have been shown to respond to tumor radiotherapy through inflammatory signal activation. Research has revealed that methyltransferase like 3 (METTL3) was related to m6A of circCUX1 in Hypopharyngeal squamous cell carcinoma (HPSCC), which was favorable for circCUX1 expression. In radiation-resistant HPSCC samples, circCUX1 was higher and negatively correlated with Caspase1. Caspase1 inhibition reduced the release of inflammatory factors IL-1β and IL-18, conferring tolerance to radiotherapy in HPSCC (87). Silencing circTUBD1 in irradiated hepatic stellate cells (HSC) decreased the release of the pro-inflammatory cytokines IL-1b, IL-6, thereby reducing radiation-induced liver disease. The mechanism showed that circTUBD1 adsorbed miR-146a-5p to regulate the expression TLR4, IRAK1, TRAF6, and pNF- κB (88). Understanding how radiotherapy affects inflammation is critical if we are to effectively modulate these cytokines to benefit oncology treatments.

Hypoxia is a classic feature of malignant tumors. Rapid proliferation of tumor cells will accelerate the consumption of oxygen. Under hypoxic conditions, tumor cells secrete a variety of vascular growth factors to promote the formation of abnormal blood vessels. In parallel, the invasive and metastatic ability of tumor cells are further improved. Eventually, the malignant degree of the tumor is further increased, resulting in the tumor cells being counteractive to treatment. Hypoxia inducible factor-11α (HIF-1α) is the key controller in tumor hypoxic microenvironment. Su demonstrated that HIF-1α was positively correlated with circDENND2A which was essential for hypoxia-induced migration and invasion of glioma cells (89). Yang found circRNA cZNF292 was hypoxia-responsive, and its expression gradually increased with the extension of hypoxia culture time. Highly expressed circRNA ZNF292 represented radioresistance in hepatoma cell (77).

These findings provide important information to better understand the hidden aspects of circRNAs and TME. However, research on the role of circRNAs in the TME related to radiotherapy resistance is in its infancy. A series of issues such as the impact of circRNAs on other cells in the microenvironment and how the TME in turn affects circRNAs remain to be resolved.

### Roles of Exosomal circRNAs in Cancer Radiotherapy

Exosomes are derived from endolysosomal microparticles and are released by fusion with multivesicular endosomes (MVEs) (90). As important bridges for intercellular communication between cells, exosomes are naturally shed by various types of cells and circulate in biofluids such as blood, saliva, urine, as well as cerebrospinal fluid, ascites (91), and carry organ-specific bioactive molecules, including proteins, nucleic acids, growth factor, lipids, and non-coding RNAs **(** **Figure 3** **)**.

Radiation affects the content and prosperity of exosomes, as well as their biological functions. There is significant growing data to show that radiation-derived exosomes accelerate tumor progression and decrease the curative rate, and is emerging as an increasingly pivotal field in the clinic. For instance, circRNAs isolated from extracellular vesicles were markedly diverse between radioresistant glioma cells and the control group, of which RNA−sequencing and bioinformatics identified 63 upregulated circRNAs and 48 downregulated circRNAs. Chen et al. identified exosomal circRNAs expression profiles of pancreatic cancer cells upon radiation, and found that circ_0002130 was highly expressed in irradiated mice plasma, and facilitated tumor cells proliferation by accelerating DNA damage repair. This effect was dependent on the interaction between circ_0002130, miR_4482-3p and targeted NBN gene (92). NBN is a member of the MRN complex family and is crucial in sensing of DNA strand break and checkpoint activation (93) (**Figure 3**). However, the above-mentioned regulation mechanism is only a prediction of the bioinformatics network and the underlying mechanism has yet to be verified by experiments. Meanwhile, the molecule has not been validated on clinical specimens. Future in-depth clinical trials will further strongly support their research. Zhao et al. found that circATP8B4 and downstream molecule miR−766 might be the decisive components in adjusting radiotherapy in glioma cells. Interestingly, circATP8B4 stems from EVs of radioresistant glioma cells and can be transferred to adjacent cells, thus promoting radioresistance of normal glioma (94). CircKIRKOS-71 and KIRKOS-73 are derived from endothelial cell exosomes and exhibit varying degrees of responsiveness following radiation in different cell lines, with a time-dependent effect. After receiving 0.25 Gy and 2.5 Gy for 24 h, KIRKOS-71 and KIRKOS-73 were down-regulated in neuroblastoma cells exosomes. In contrast, in the osteosarcoma cell line U2OS, elevated transcript levels were measured in exosomes irradiated for 24 h with low-dose irradiation and 4 h of medium-dose irradiation (95). This provides a theoretical basis for the diagnostic strategy of exosomal circRNAs in clinical radiation. The expression level of exosomal circ_0067835 was upregulated in CRC patients after radiation, while knockdown diminished CRC deterioration and enhanced radiosensitivity by down-regulating insulin-like growth factor receptor (IFG1R) expression through decoying miR-296-5p (96). However, the real mechanism between miR-296-5p/IFG1R and circ_0067835 in CRC radiotherapy is unclear. For example, it is unknown whether PI3K/Akt and MAPK pathways were mediated by IFG1R.

Of note, the mechanism of action of exosomes is different in various conditions. The studies by Dai (97) and Wang (96) indicated that aberrant expression of exosomes was linked to carcinogenesis, malignant behavior and radioresistance of glioblastoma. Nevertheless, Farias (98) proposed that the exosomes released by irradiated mesenchymal stem cells have systemic effects and could delay the growth and metastasis of melanoma. In a word, clearer mechanisms of circRNAs and further understanding of exosomes in radiation are needed.

## The Clinical Application of circRNAs in Radiotherapy

Data from previous studies proposed that circRNAs exhibited tissue and organ specificity, and were dysregulated in numerous human cancers. Intriguingly, the expression profiles of circRNAs are also divergent in different stages within the same type of tumors, as well. Owing to the signatures of stability, specificity and availability, circRNAs have irreplaceable potential in clinical oncology strategies. At this stage, the clinical research of circRNAs in tumor radiotherapy mainly focuses on the following two strategies: (1). Different expression patterns of circRNAs in patients, that is, to analyze the differential expression of circRNAs in radiation-sensitive or radiation-resistant patients, as the object of further research (99–101). For example, the expression of circRNA_0000285 and circCUX1 in radiotherapy-resistant patients is higher than that in radiosensitive patients. Therefore, the feasibility of radiotherapy can be predicted by analyzing circRNA expression. (2). Analysis of circRNAs expression level after irradiation (62, 102, 103). With the help of high-throughput sequencing, Yu et al. analyzed 153 differentially expressed circRNAs between control group and HeLa cells accepted radiotherapy, identified 76 increased circRNAs and 77 decreased (104). Similarly, compared with the parental cell line, 57 circRNAs were elevated and 17 circRNAs decreased in radiation-resistant esophageal cancer cells identified by cirRNA microarray (76). These altered circRNAs may participated in the regulation of radiation resistance at the transcriptional level. The above research strategies both suggest that circRNAs have differential expression profiles in radiotherapy. These circRNAs are expected to become sensitive indicators in the treatment of cancer patients and provide a basis for clinicians to formulate individualized treatments.

### CircRNAs Are Expected to Become a Targeted Therapy Point in Radiation

Radiosensitizers are drugs or methods that are used simultaneously with radiotherapy to increase the sensitivity of radiotherapy, by regulating signal pathways or target molecules involved in radioresistance. The above-mentioned studies indicate that circRNAs have the potential to become new clinical radiosensitizers. The following three methods can be utilized to regulate the radiation response involving circRNAs, thereby improving the efficacy of RT: 1. For circRNAs that act as suppressors (radiotherapy tolerance) or promotors (radiotherapy sensitivity) **(** **Table 1** **)**, regulating their expression level seems to be an effective strategy. Circ_0055625 knockdown sensitized colon cancer to irradiation and inhibited tumor malignancy (109). Silencing circRNA_000543 improved radiosensitivity in NPC, impeding proliferation and invasion, while promoting apoptosis (99). On the contrary, circ_000128 showed low expression in NSCLC cells and tissues. Circ_0001287 overexpression could up-regulate PTEN to repress the multiplication, metastasis, and radioresistance of NSCLC cells through endogenous competition with miR-21 (107). 2. In addition to directly interfering with circRNAs, focusing on the targets or pathways is another idea of improving radiotherapy. It has been demonstrated that circABCB10 and circPITX1 participated in radiation response *via* glycolysis. Hence, the glycolytic inhibitor 2-deoxy-D-glucose (2-DG) constrains radiation resistance caused by these two circRNAs. Application of the autophagy inhibitor chloroquine reversed the differential expression of LC3II/I and p62 in LoVo/R cells, and the radiotherapy sensitization ratio was higher than before. It also antagonized the autophagy of circBANP in the colon cancer RT. CircRNA_000543 could regulate the radioresistance of NPC cells by targeting the miR-9/platelet-derived growth factor receptor B(PDGFR)axis. The PDFGRB inhibitor imatinib sensitized radioresistance in NPC cells (99). 3. Some well-known bioactive compounds can enhance radiosensitivity by modulating circRNAs. Curcumin, a traditional Chinese medicine herb, has been widely studied in the field of cancer treatment in recent years. Zhu et al. discovered curcumin restored the increased expression of circRNA_102115 in NPC cells after irradiation. Adding curcumin during radiation could recover radiosensitivity through the circRNA_102115/miR-335-3p/MAPK1 axis (84).

**Table 1:** The dysregulated circRNAs in radiotherapy of malignant tumors.

System	Cancer	Type of circRNAs	Dysregulation (after RT)	Target/pathway	Functions	Impact on RT	Ref
Respiratory system	NPC	circRNA_000543	up (compare to radiosensitive)	miR-9/PDGFRB	apoptosis, invasion, proliferation	radioresistant	(99)

		exosomal circMYC	up (compare to radiosensitive)	miR-20b-5p (predict) let-7e-3p (predict)	proliferation	radioresistant	(105)
		circRNA_0000285	up (compare to radiosensitive)	–	–	radioresistant	(100)

		circRNA_001387	up	–	–	radioresistant	(106)
		circFIP1L1	down	miR-1253/SFN	cell cycle	radiosensitivity	(71)
		circRNA_102115	up	miR-335-3p/MAPK1	cancer stem cell	radioresistant	(84)
	HPSCC	circCUX1	up (compare to radiosensitive)	Caspase1	inflammatory factors	radioresistant	(87)

	NSCLC	circ_0086720	up	miR-375/SPIN1	cell apoptosis, DNA damage repair	radioresistant	(54)
		circMTDH.4	up (compare to normal)	miR‐630/AEG‐1	proliferation, migration, invasion, apoptosis	radioresistant	(79)

		circ_0001287	down	miR-21/PTEN	proliferation, migration	radiosensitivity	(107)
		circPVT1	up	miR-1208 PI3K/AKT/mTOR	apoptosis	radioresistant	(60)
Digestive system	OSCC	circATRNL1	down	miR23a-3p/PTEN	apoptosis, cell cycle	radiosensitivity	(62)

	Esophageal cancer	circPRKCI	up (compare to normal)	miR-186-5p/PARP9	cell cycle, colony formation, cell viability	radioresistant	(74)

		circVRK1	down	miR-624-3p/PTEN/ PI3K/AKT pathway	apoptosis	radiosensitivity	(61)

		hsa_circ_0000554	up	miR-485-5p/Fermt1	proliferation, migration, invasion, apoptosis	radioresistant	(108)

		circRNA_100367	up	miR-217/wnt3	proliferation, migration	radioresistant	(78)

	Liver cancer	circRNA ZNF292	up (under hypoxia)	SOX9 protein	proliferation, angiogenesis	radioresistant	(77)
				Wnt/β-catenin			
		circTUBD1	up	miR-146a-5p	inflammatory factors	radioresistant	(88)
				TLR4 Pathway			
	Colorectal cancer	exosomal circ_0067835	up	miR-296-5p/IGF1R	proliferation, apoptosis, cell cycle	radioresistant	(96)


		circRNA CBL.11	up	miR-6778-5p/YWHAE	proliferation, apoptosis	radioresistant	(58)
				P53 signaling pathway			
		circ_0055625	up	miR-338-3p/MSI1	proliferation, migration, invasion, apoptosis	radioresistant	(109)


		hsa_circ_0001313	up	miR-338-3p	cell viability, colony formation	radioresistant	(110)
		circBANP	up	miR-338-3p	autophagy	radioresistant	(66)


Urinary system	Prostate Cancer	circ_0062020	down	miR-615-5p/TRIP13	proliferation, metastasis, apoptosis, colony formation	radioresistant	(103)


		circZEB1	up	TR4-mediated QKI/miR-141-3p/ZEB1	DNA damage repair	radioresistant	(51)
		circ_CCNB2	up (compare to normal)	miR-30b-5p/KIF18A	autophagy	radioresistant	(68)

Central nervous system	Glioma	circCPA4	up (compare to normal)	miR-760/MEF2D	proliferation, apoptosis, migration, invasion	radioresistant	(111)

		circ_VCAN	down	miR-1183	proliferation, migration, invasion, apoptosis	radioresistant	(102)


		circPITX1	up (compare to normal)	miR-329-3p/NEK2	glycolysis	radioresistant	(82)

		circular AKT3	down (compare to normal)	AKT3-174aa/PDK-1	proliferation, apoptosis	radiosensitivity	(55)
				PI3K/AKT signal			
		circ-METRN	up (in low-dose radiation)	miR-4709-3p/GRB14/PDGFRα	DNA damage repair	radioresistant	(53)

Endocrine system	Cervical cancer	hsa_circ_0009035	up (compare to radiosensitive)	miR-889-3p/HOXB7	proliferation, apoptosis, migration, cell cycle	radioresistant	(101)

	Pancreatic cancer	circ_0002130	up	miR_4482-3p/NBN	DNA damage repair	radioresistant	(92)
	Breast cancer	circABCB10	up (compare to normal)	miR-223-3p/PFN2 axis	glycolysis	radioresistant	(81)

Numerous experiments *in vivo*/vitro have shed light on the interference or overexpression of circRNAs that can antagonize tumor progression. Hence, a series of targeting circRNAs techniques as therapeutic approaches may have clinical value. The loss-of-function strategy of circRNAs includes CRISPR/Cas9 or CRISPR/Cas13-mediated knockdown, and the RNA interference (RNAi) mechanism based on short interfering RNA (siRNA) or short hairpin RNA (shRNA). In addition to lentiviral or adenoviral vectors, chemical synthesis and purification are used to overexpress circRNAs. The delivery of target circRNAs *in vivo* focuses on exocrine and nanoparticles (112). For instance, in a mouse model of nonalcoholic steatosis, the author injected high-fat diet mice with nanoparticles encapsulated circRNA SCAR, which notably alleviated symptoms of liver cirrhosis (113). Recently, some scientists synthesized novel tools named small circular interfering RNAs (sciRNAs) which are sense strands functionalized with GalNAc ligand then annealed into antisense strands after chemical modification. The effect of sciRNAs *in vivo/vitro* is equivalent to that of clinically used siRNA (114). However, the targeted therapy of circRNAs is still in its infancy. The safety and efficacy of circRNA-based therapeutics are yet to be ensured.

### CircRNAs Are Used to Predict the Effect of Radiotherapy

Predicting tumor response to radiotherapy, recurrence, and prognosis are necessary to improve the cure rate of patients. In order to study the clinical value of circRNAs in predicting NPC radiotherapy, Shuai detected the expression of circRNA_001387 in RT patients and cell lines. The patients who were tolerant to RT had higher expression of circRNA_001387 than those who were sensitive to radiotherapy. Surprisingly, the expression of circRNA_001387 in cells was apparently increased with the frequency of irradiation enforced (106). Highly expressed exosomal circMYC is related to clinicopathological parameters in NPC, such as survival rate and recurrence. Gain-functional experiments indicated that overexpression of circMYC reduced radiosensitivity, suggesting that circMYC might afford vital characteristics as a target for radiotherapy efficacy (105), to some extent. Circ_0062020 functioned as a competing endogenous RNA to restrain the radiosensitivity by modulating miR-615-5p/TRIP13 axis in prostate cancer (PCa) cells. The upregulation of circ_0062020 was detected in PCa tissues including radiosensitive and radioresistant tissues in contrast to adjacent normal tissues, especially in radioresistant tissues (103). Chen et al. found circRNA_000543 expressed at high levels in radioresistant NPC compared to radiosensitive samples (99). CircRNAs isolated from extracellular vesicles were markedly diverse between radioresistant glioma cells and the control group, of which RNA−sequencing and bioinformatics analyzed 63 upregulated circRNAs and 48 downregulation (94). It is becoming increasingly difficult to ignore the significant roles of circRNAs play in radiotherapy. Therefore, if further validated in larger-scale clinical research, circRNAs are expected to be complementary to traditional clinical assessment indicators.

## Conclusions and Future Perspectives

With the development of bioinformatics and experimental techniques, the characteristic and functions of circRNAs have been widely elaborated. Particularly in the field of cancer, these remarkable new molecules exert their unparalleled status in all aspects of tumor biology. To data, only a few studies have been carried out on the actions of circRNAs in RT responses. In this article, we summarized the different underlying mechanisms involved in the multiple aspects of cellular response to IR. CircRNAs are a double-edged sword in radioresistant tumors, as they can not only promote radioresistance but also inhibit radioresistance. Functional experiments have demonstrated that regulation of circRNAs can obviously benefit radiotherapy, implying the great potential of targeting circRNAs. Research on the regulatory mechanisms of circRNAs in tumor radiation therapy will be the future research trend.

At present, the research on the role of circRNAs in tumor radiotherapy is still in its infancy, and plenty of aspects regarding their regulatory mechanisms remain undefined. Currently, the mainstream research on radiotherapy-related circRNAs is still focused on ceRNAs, and mechanisms to determine other models are strongly required. In this review, it was evident that most circRNAs acted as miRNA sponges to activate or inhibit signal pathways, except for circCUX1 (87) and circRNA ZNF292 (77), which functioned as regulatory proteins, and circular AKT3 (55), which played translational functions. We believe that further research can focus more on other molecular mechanisms, such as post-transcriptional modifications and translation machinery. A thorough understanding of the molecular mechanism of circRNAs will help to identify novel and effective diagnostic and therapeutic targets.

**Figure 4:**
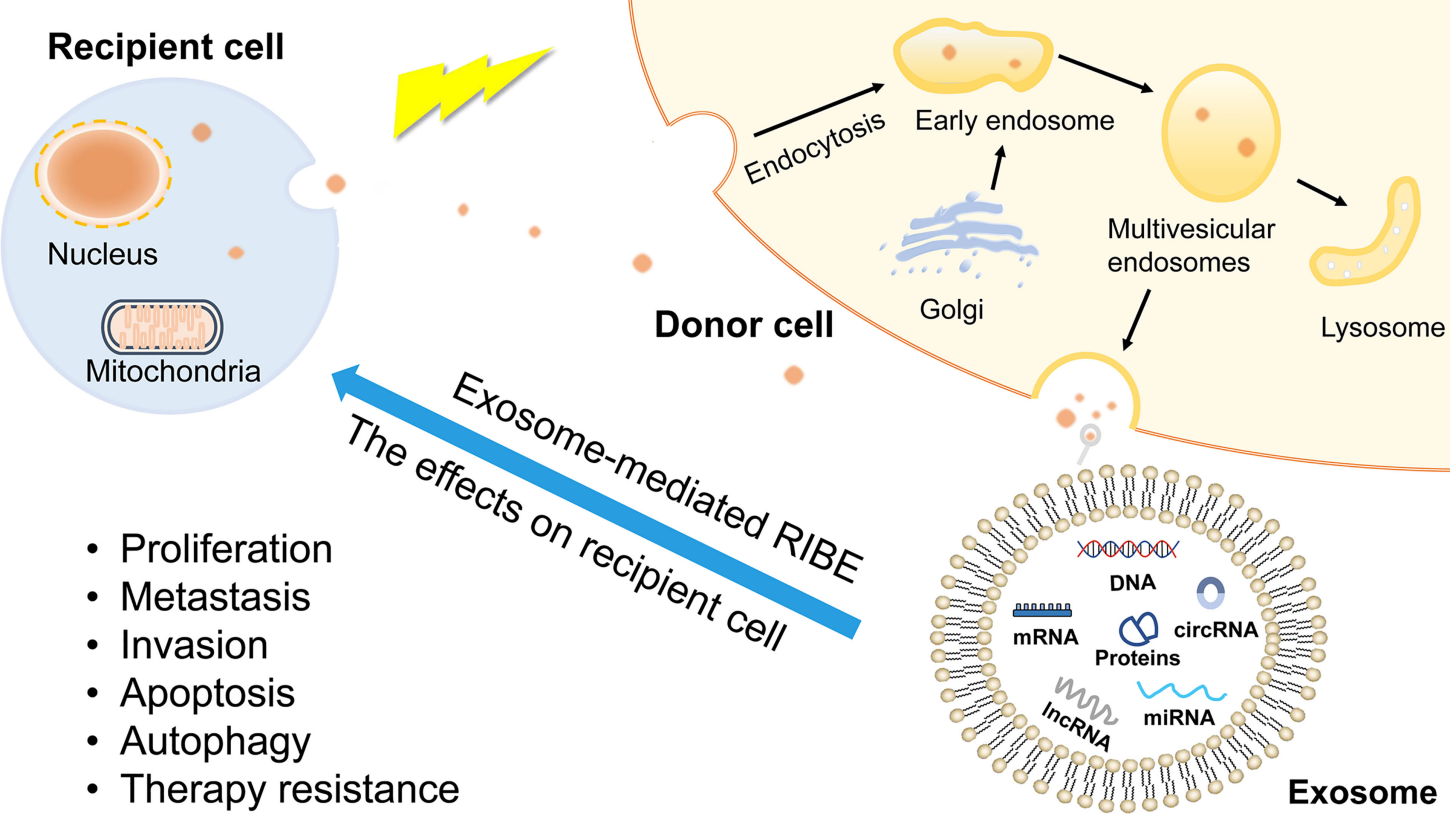
The production of exosomes and exosome-mediated RIBE. Exosomes are derived from endolysosomal microparticles and are released by fusion with MVEs. The contents of exosomes include proteins, nucleic acids, growth factor, lipids, non-coding RNAs. RIBE is mediated by gap junction or substances released into extracellular environment. As bystander effect mediators, exosomes induce recipient cells present the same outcomes as directly exposed cells, such as proliferation, metastasis, therapy resistance.

In addition to the direct effects of radiation on irradiated cells, studies have found that non-irradiated cells are also indirectly affected by radiation, a process known as radiation-induced bystander effect (RIBE) (115). RIBE is a major factor in determining the success of radiotherapy, not only because of the damage to irradiated cells, but also because it can induce cancer cells to become resistant to radiotherapy (116). Exosomes are important mediators in the bystander effect **(** **Figure 4** **)**. It has been reported that miRNAs in exosomes can alter radiotherapy efficacy through RIBE (117, 118). To the best of our knowledge, circRNAs research on the benefits of RIBE has not yet been reported so far. Therefore, we infer that circRNAs can also induce RIBE through exosomes. Further research may help to discover the association between circRNAs and RIBE, and establish early interventions against RIBE to improve the efficacy of radiotherapy.

As of now, no circRNA has really been applied in clinical practice. Targeting circRNAs as a therapeutic strategy remains obstacles and bewilderments. Meanwhile, the studies of circRNAs in radiotherapy are limited to only a few solid tumors. To date, there have been no reports on the RT of hematological malignancies. The potential clinical value of circRNAs in tumor progression and drug resistance has been highlighted. Expanding the research field of circRNAs will undoubtedly broaden our horizon. These deficiencies will continue to be overcome in the future, and the value of circRNAs as biomarkers in radiotherapy is worthy of attention.

## Author Contributions

BW and HY designed the review. XW and JW collected the literature and wrote the manuscript. LW and WY revised the manuscript. All authors read, reviewed, and approved the final manuscript.

## Funding

This study was supported by the National Natural Science Foundation of China (grant no.81702078), the Natural Science Foundation of Jiangsu Province (grant no.BK20170356); Suzhou Science, Education and Health Youth Science and Technology Project (grant no.KJXW2020019); Suzhou City, the seventh batch of “Gusu Health Talent Plan” youth top talent project; Support for the project of nuclear technology medical application supported by discipline construction (grant no.XKTJ-HRC2021001); The Project of State Key Laboratory of Radiation Medicine and Protection, Soochow University (grant no.GZK1202137); Suzhou Science and Technology Project (grant no.SKY2021045, SKJY2021097); Maternal and Child Health Association Project of Jiangsu province (grant no.FYX202123).

## Conflict of Interest

The authors declare that the research was conducted in the absence of any commercial or financial relationships that could be construed as a potential conflict of interest.

## Publisher’s Note

All claims expressed in this article are solely those of the authors and do not necessarily represent those of their affiliated organizations, or those of the publisher, the editors and the reviewers. Any product that may be evaluated in this article, or claim that may be made by its manufacturer, is not guaranteed or endorsed by the publisher.

## Abbreviations

DDR, DNA damage repair; EMT, Epithelial–mesenchymal transition; ICFs, Intron-containing fragments; RBPs, RNA-bind proteins; ADAR1, Adenosine deaminase acting on RNA 1; EGFR, Epidermal growth factor receptor; ATM, ataxia telangiectasia-mutated; ATR, ATM and Rad3 related protein; DNA-PK, DNA-dependent protein kinase; H2AX, H2A histone family member X; ZEB1, Zinc finger E-box binding homeobox 1; PDGFRα, Platelet derived growth factor α; PTEN, phosphate and tension homology on chromosome ten; CDKs, cyclin-dependent kinases; MVEs, multivesicular endosomes; RIBE, Radiation-induced bystander effect; IFG1R, Insulin-like growth factor receptor; IR, irradiation; RT, radiotherapy; CSCs, Cancer stem cells; APC, Adenomatous polyposis coli; Axin, axis inhibition; TME, Tumor microenvironment; HIF-1α, Hypoxia inducible factor-11α (HIF-1α); NPC, Nasopharyngeal Carcinoma; HPSCC, hypopharyngeal squamous cell carcinoma; NSCLC, non-small cell lung cancer; OSCC, oral squamous cell carcinoma; GBM, glioblastoma multiforme; 2-DG, 2-deoxy-d-glucose.
